# Natural history of Myhre syndrome

**DOI:** 10.1186/s13023-022-02447-x

**Published:** 2022-07-30

**Authors:** David Dawei Yang, Marlene Rio, Caroline Michot, Nathalie Boddaert, Wael Yacoub, Nicolas Garcelon, Briac Thierry, Damien Bonnet, Sophie Rondeau, Dominique Herve, Stephanie Guey, Francois Angoulvant, Valerie Cormier-Daire

**Affiliations:** 1 Centre de Recherche Des Cordeliers, INSERM UMRS 1138 Team 22, Université de Paris, 75006 Paris, France; 2 Pediatric Emergency Department, AP-HP, Hôpital Universitaire Necker-Enfants Malades, 75015 Paris, France; 3 Université de Paris, Institut IMAGINE, Developmental Brain Disorders Laboratory, INSERM UMR1163, 75015 Paris, France; 4 Departement of Medical Genetics, AP-HP, Hôpital Universitaire Necker-Enfants Malades, 75015 Paris, France; 5 Université de Paris, Institut IMAGINE, Molecular and Physiopathological Bases of Osteochondrodysplasia, INSERM UMR1163, 75015 Paris, France; 6 Paediatric Radiology Department, AP-HP, Hôpital Universitaire Necker Enfants Malades, 75015 Paris, France; 7 Université de Paris, Institut IMAGINE, INSERM1163, 75015 Paris, France; 8 Université de Paris, Institut IMAGINE, Data Science Platform, INSERM UMR1163, 75015 Paris, France; 9 Department of Pediatric Otolaryngology-Head and Neck Surgery, AP-HP, Hôpital Universitaire Necker – Enfants Malades, 75015 Paris, France; 10 Université de Paris, Human Immunology, Pathophysiology, Immunotherapy/HIPI/INSERM UMR976, Stem Cell Biotechnologies, 75010 Paris, France; 11 M3C-Paediatric Cardiology, AP-HP, Hôpital Universitaire Necker Enfants Malades, 75015 Paris, France; 12 Department of Neurology, AP-HP Nord, Referral Center for Rare Vascular Diseases of the Brain and Retina (CERVCO), DHU NeuroVasc, INSERM U 1161, 75010 Paris, France; 13 Department of Neurology, AP-HP, Hôpital Lariboisière, UMR-S1161, 75010 Paris, France

## Abstract

**Background:**

Myhre syndrome (MS) is a rare genetic disease characterized by skeletal disorders, facial features and joint limitation, caused by a gain of function mutation in *SMAD4* gene. The natural history of MS remains incompletely understood.

**Methods:**

We recruited in a longitudinal retrospective study patients with molecular confirmed MS from the French reference center for rare skeletal dysplasia. We described natural history by chaining data from medical reports, clinical data warehouse, medical imaging and photographies.

**Results:**

We included 12 patients. The median age was 22 years old (y/o). Intrauterine and postnatal growth retardation were consistently reported. In preschool age, neurodevelopment disorders were reported in 80% of children. Specifics facial and skeletal features, thickened skin and joint limitation occured mainly in school age children. The adolescence was marked by the occurrence of pulmonary arterial hypertension (PAH) and vascular stenosis. We reported for the first time recurrent strokes from the age of 26 y/o, caused by a moyamoya syndrome in one patient. Two patients died at late adolescence and in their 20 s respectively from PAH crises and mesenteric ischemia.

**Conclusion:**

Myhre syndrome is a progressive disease with severe multisystemic impairement and life-threathning complication requiring multidisciplinary monitoring.

## Introduction

Myhre syndrome (MS) (MIM#139210) is a rare developmental disorder reported for the first time in 1981 [1]. The main clinical features are short stature, neurodeveloppemental deficiency, facial dysmorphism (prognathism, short palpebral fissure, maxillar hypoplasia), deafness, muscular hypertrophia, joint limitations and skeletal features such as short extremities, enlarged vertebral pedicles and thickened calvarium. Heterozygote mutations altering the Ile500 residue of *SMAD4* have been reported in 2011 as the disease mechanism [2]. Shortly after, another mutation affecting residue Arg496 was identified in 2014 [3].

Since the canonical description of MS, about 90 patients have been published in the literature, including 70 patients with molecular confirmation [1–22]. Reported cases showed that consistent features are short stature, facial dysmorphism, thickened skin, joint limitation and muscular hypertrophia. Less frequent features have been also reported with a wide range of clinical presentation [4], including life-threathing complications such as pericarditis and laryngotracheal stenosis [23]. Nevertheless, the natural history of MS remains incompletely understood.

Natural history studies of rare genetic diseases encounter two major issues: a low number of cases in contrast with the heterogeneity of clinical presentation [24]. An exhaustive description of the entire clinical spectrum leads to a long and complex data collection. However, the Necker-Enfants malades University Hospital (NEM) hosts the French national reference center for rare skeletal dysplasia (RC RSD) which provides longitudinal follow-up of patients with MS. In addition, the hospital has a clinical data warehouse for secondary use of electronic health record. The aim of our study was therefore to describe the natural history of Myhre syndrome.

## Materials and methods

We set a monocentric retrospective longitudinal study. We included patients with MS with molecular confirmation of mutation in *SMAD4* from the RC RSD registry. The exclusion criteria were patients with clinical criteria for MS without *SMAD4* confirmation. We built a standardized clinical report form from the online databases of OMIM (MIM number # 139210) [25, 26] and ORPHADATA (ORPHA number 2588) [27] which provides a phenotypic description of the genetics disease with the Human Phenotype Ontology (HPO) thesaurus [28–31].

Data collection was carried out in 3 steps: (1) From the paper medical records of the patients. (2) From electronic health record by using Dr. Warehouse (DrWH), a clinical data warehouse operating software focused on free text analysis [32]. (3) All medical imaging documents and patients photographies were reviewed respectively with the medical imaging department and clinical genetics department. Management of missing data was ensured by chaining the information from the different data sources.

Statistical analyzes were performed with R^®^ v4.0.4. Qualitative data are reported as counts and percentages and quantitative data as median and interquartile values. Data collection, storage and secondary use of the hospital data warehouse with DrWH have been approved by the French Insitutionnal Review Board Il-de-France II (IRB registration number 00001072) registered under reference 2016-06-01.

## Results

### Demographic characteristics

We assessed 12 patients with MS and a heterozygous and de novo* SMAD4* mutation. The median follow-up period was 7 years (Q1 = 3 years; Q3 = 12 years). Six cases were already reported by Michot et al. [4], but their natural history was not specified and the six others are new cases. *SMAD4* mutations were c.1498A > G (p. (Ile500Val)) for 9 patients, c.1499T > C (p. (Ile500Thr)) for 2 patients and c.1486C > T (p. (Arg496Cys)) for one patient.

The median age at diagnosis suspicion was 12 years (Q1: 6 years; Q3: 14 years) and median age at molecular confirmation was 14 years (Q1: 8 years; Q3: 22 years). The study population had a sex ratio of 1/3 (Male = 3; Female = 9). We reported three deaths, all of them concerning patients with Ile500Val mutations. Two patients died in late adolescence respectively from pulmonary hypertension crisis and mesenteric infarction; the third patient died in toddler from severe esophageal atresia. The median age of surviving patients is 22 y/o (Q1: 18 y/o; Q3: 23 y/o) (Table 1).

**Table 1:** Natural history of Myhre syndrome: approximative age of onset of main clinical features

Age of onset	Infancy < 12 months	Toddler 12 months–2 y/o	Early childhood 2–5 y/o	Middle childhood 6–11 y/o	Adolescence 12–18 y/o	Adult > 18 y/o
Growth	Intra-uterine growth retardation	Failure to thrive		Muscular hypertrophy Short stature	Overweight	
Specific facial feature				Prognathism Maxillar hypoplasia Narrow palpebral fissure Thin upper lip		
Specific skeletal feature		Extremities abnormalities		Cone shaped epiphyses Thickened calvarium Enlarged vertebral pedicles Joints limitations		
Skin				Stiff and thick skin		
Cardiovascular	Congenital heart defect		Pulmonary arterial hypertension	Pericarditis Vascular stenosis		
Neurologic		Neurodevelopmental delay	Intellectual disability	Behavioral disorder		
Respiratory				Laryngotracheal stenosis Pleural effusion		
Gastro-enterology	Esophageal atresia	Digestive stenosis				
Sensory system			Hearing impairment Refractive disorders			
Endocrine				Precocious puberty		Infertility Diabetes mellitus
Immunity			Hypogammaglobulinemia			

### Clinical characteristics and natural history

#### Growth

Intra uterine growth retardation (IUGR) and failure to thrive were reported in all cases (12/12). IUGR were moderate with median at − 2 SD (Q1: − 3 SD; Q3: − 2 SD) but became more severe in post-natal life with median height of − 3.5 SD (Q1: − 4 SD; Q3: − 3 SD). We noticed that one patient received growth hormone treatment, without any benefit. From 6 y/o, some patients had specific gestalt with muscular hypertrophy (9/12; 75%). Overweight was reported in 4 patients with an onset in early adolescence.

#### Facial features

The most frequent facial features were prognathism (11/12; 92%), maxillar hypoplasia (9/11; 82%), narrow palpebral fissures (9/12; 75%), small ears (8/11; 73%), broad nasal bridge (6/9; 67%), thin upper lip (6/10; 60%), short philtrum (7/12; 58%), small mouth (5/12; 42%), thick eyebrows (3/9; 33%) and a short neck (3/9; 33%). These features were identified in medical record mainly at 6 y/o (Q1: 3 y/o; Q3: 7 y/o).

#### Skeletal features

Skeletal X ray imaging were available for 9 of 12 patients. The most frequent characteristics were found at the extremities of limbs and were identified from the first years of life, with brachydactyly (11/11; 100%) and small hands (8/8; 100%). Clinodactyly (4/8; 50%), camptodactyly (2/8; 25%) or toes syndactyly 2–3 (2/9; 22%) were also reported. More detailed radiological analysis identified cone-shaped epiphyses in phalanges in 50% of patients (4/8). Axial skeleton presented specific features and were mainly identified at the age of 6 y/o, such as thickened calvarium (5/7; 71%), enlarged vertebral pedicles (7/10, 70%), iliac wings hypoplasia (5/9; 56%); broad ribs (4/9; 44%). We reported also peculiarities in limbs such as short long bones (3/6; 50%) and short femoral necks (5/9; 56%). Patients developed joint limitation in 89% of cases (8/9), with a median age at 6 y/o (Q1: 3 y/o; Q3: 8 y/o), starting from small joints, then continuing to a global impairment.

#### Sensory system

Hearing impairment was reported in our study in 58% (7/12) of cases and was detected from 2 years old. Etiologies were conductive (n = 3), perceptive (n = 2) or mixed (n = 2). Visual impairment was observed in 9 of 12 patients (75%), concerning mainly refractive error such as hyperopia (n = 6) and sometimes associated with strabismus (5/11; 45%). We also noticed cataract in 3 patients.

#### Skin

Thickened and stiff skin was observed in 8 of 12 patients (67%), with an onset in middle childhood.

#### Cardiovascular (congenital and acquired)

Congenital heart defects were common in MS (n = 7, 58%); three patients had a persistent arterial duct which required surgical closure. Two patients had a Shone's complex characterized by supravalvar mitral ring, parachute mitral valve, subaortic stenosis, and aortic coarctation. These two patients underwent mitral valve replacement. Isolated defect such as ventricular septal defect (n = 1) and atrial septal defect (n = 1) and aortic stenosis (n = 3) were also observed. Pericarditis was observed in 2 patients, diagnosed at early and middle childhood respectively, with favorable outcome.

Eight patients had pulmonary pressure assessment by echocardiography and 3 patients were explored by right heart cath. Five of 8 patients (63%) had pulmonary hypertension (PH) with mean pulmonary artery pressure 40–50 mmHg. Etiologies were various including unilateral stenosis of the left pulmonary artery (n = 1), postcapillary PH caused by mitral valve disease in the context of a Shone's complex (n = 1), and multifactorial such as related to pulmonary sequestration (n = 1) and of respiratory origin with chronic obstruction of the airways (n = 1). The age of onset of PH were noted at early childhood for patients with Shone's complex and from early adolescence for other causes. As described earlier, one patient died at late adolescence after a pulmonary hypertensive crisis.

**Fig. 1:**
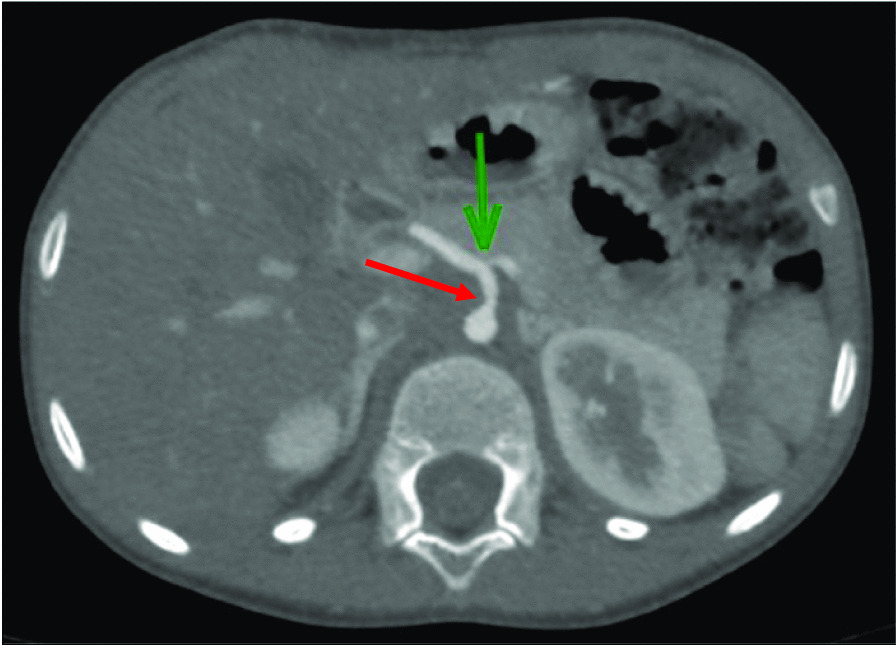
Computerized tomography angiography. *These images correspond to patient 5*. Transverse reconstruction centered on the celiac trunk: Stenosis of the initial segment of the splenic artery (top arrow) and the post ostial segment of the celiac trunk (left arrow)

Vascular imaging was performed in 7 patients, including vascular ultrasonography (n = 3) and computerized tomography angiography (n = 4). Stenosis in the large and medium vessels were reported in 2 patients. One patient had stenosis of the celiac, splenic and polar branches of the renal arteries (Fig. 1). The second patient had left main carotid artery stenosis. Imaging investigations were made at middle childhood and late adolescence while patients were asymptomatic. As described earlier, one patient died at late adolescence from mesenteric infarction, without prior vascular assessment.

**Fig. 2:**
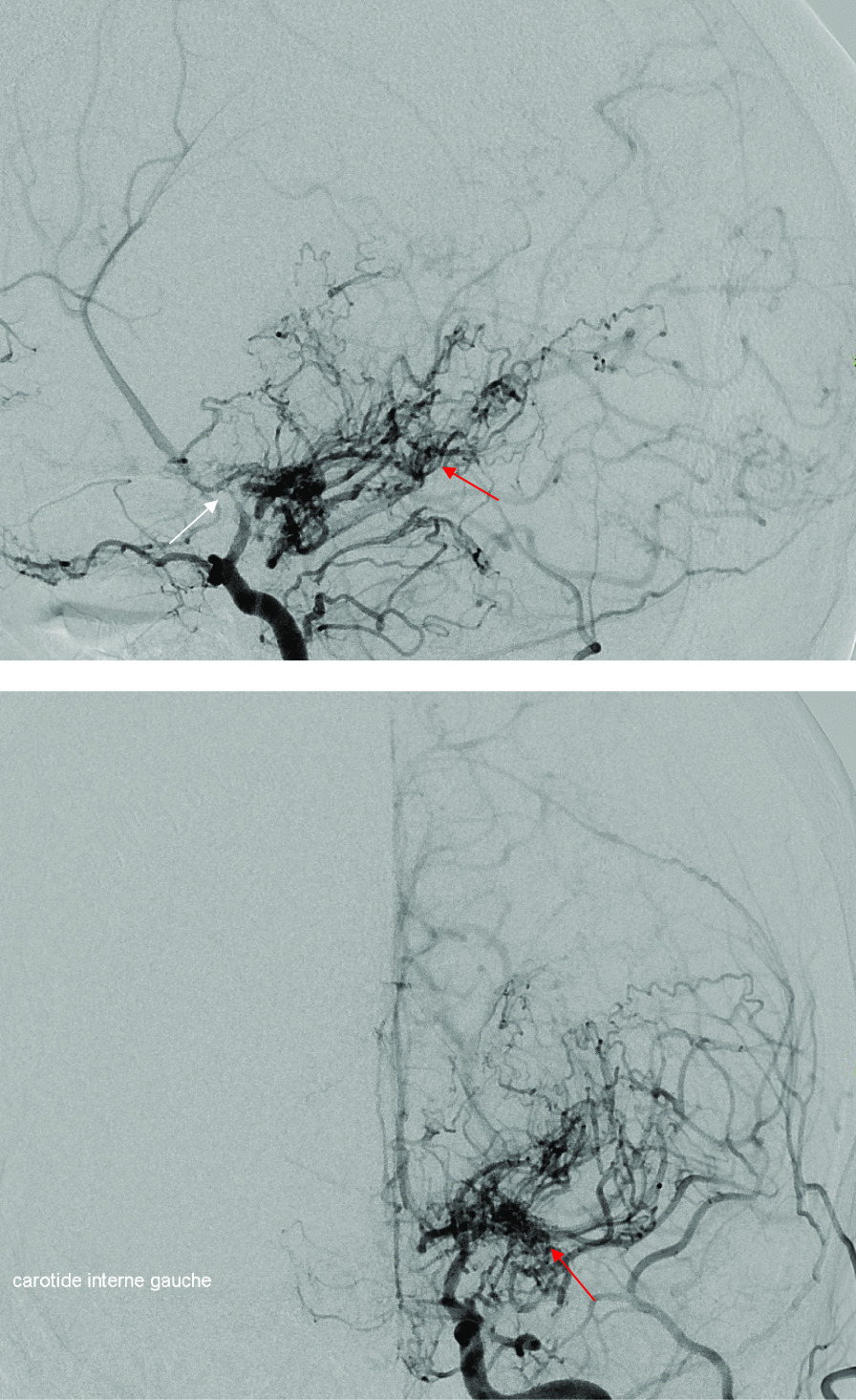
Left carotid arteriography. Profile incidence (top), frontal incidence (bottom): Stenosis of the termination of the left internal carotid artery (left arrow) with development of collateral circulation through lenticulo-striated perforating vessels, producing the specific “puff of smoke appearance”of moyamoya syndrome (right arrow). *These images correspond to patient 9*

**Fig. 3:**
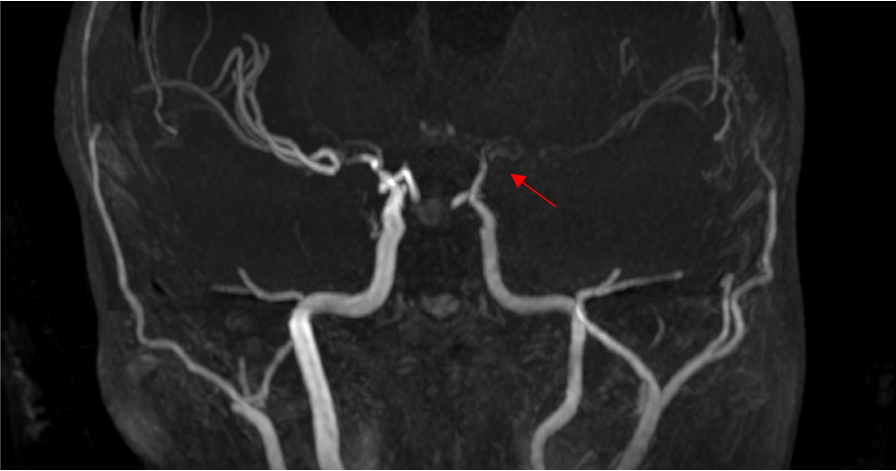
Magnetic Resonance Angiography, Time of Flight sequence. Coronal reconstruction in Maximum Intensity Projection mode: Stenosis of the end of the left internal carotid artery. *These images correspond to patient 9*

We identified one female patient with recurrent strokes in adult age. Neurovascular imaging with angiography (Fig. 2) and cerebral magnetic resonance imaging (MRI) (Fig. 3) showed a moyamoya syndrome. Consequently, brain MRI performed in 7 other patients were re-analyzed and anatomic variants of Willis polygon without pathological abnormality were found in 2 patients.

#### Neurologic and neurodevelopmental

Microcephaly at birth was observed in 4 of 10 patients and range between − 2 SD and − 3 SD. Head circumference mature in the same range of standard deviations in childhood, by contrast, other patients (n = 2) have stable macrocephaly (+ 2.5 SD).

A large majority of patients presented neurodevelopmental delay and intellectual disability (9/12; 75%) starting at early childhood; ranging from moderate to severe. Concerning schooling, information was available for 8 patients: 4 patients were in a medico-educational institute and 4 were attending conventional school with specific arrangements. Regarding the development in adulthood, the two adult patients were integrated on a socio-professional level. Behavioral disorders such as autistic-like behavior, were present at middle childhood one patient, and psychiatric manifestations with hallucinations and aggressiveness begun at early adolescence in another patient.

Brain MRI performed in 8 of 12 patients detected non-specific white matter hypersignals in 5 of 8 patients. One patient presented with idiopathic intracranial hypertension.

#### Respiratory

Two patients presented a rare form of progressive multilevel acquired laryngotracheal stenosis. A tracheostomy was required in one of them at early adolescence for symptomatic acute tracheal stenosis She also had choanal atresia. She developed recently after a subcanular stenosis which required endoscopic treatment and custom-made calibration cannula. Obstructive sleep apnea syndrome was confirmed in 4 patients. Pleural effusion was observed in 6 of 10 patients (60%), including one patient with recurrent pleural effusion. Pleural puncture was performed in 4 cases, Streptococcus pneumoniae and Haemophilus influenzae were identified in 2 patients respectively. Computed tomography scan revealed non-specific micro-calcifications in the pulmonary parenchyma in 3 patients. Three patients had medical treatment for asthma. Combination of obstructive pathologies and recurrent pleural effusion led to chronic respiratory failure in the most severe patients (n = 2) during the adolescence.

#### Gastro-intestinal

Digestive stenosis was described in two patients. We reported one patient with duodenal stenosis operated at toddler with favorable course, but as described earlier we also reported one patient with severe esophageal atresia, who had multiple operation for anastomotic stenosis with fatal issue in toddler.

#### Endocrine

Eight female patients had precocious puberty starting from the age of 8 y/o. In male patients, cryptorchidism was noted in one patient. In adulthood, one patient had ovarian insufficiency diagnosed following an infertility assessment. Diabetes mellitus was diagnosed in one adult patient.

#### Immunity

Four patients had hypogammaglobulinemia including 2 patients with a global hypogammaglobulinemia G deficiency, with clinical impact by repeated respiratory tract infections and treated with intravenous immunoglobulin cures until the end of middle childhood. The 2 other patients presented respectively a hypogammaglobulinemia A and hypogammaglobulinemia G2 and G4, without impact or need for specific treatment.

We summarize the approximative age of onset of clinical features in Table 1. The detailed phenotypic description of each patient is available and main clinical signs of our cohort are summarized in Table 2.

**Table 2:** Detailed characteristics of patients

Patient	1	2	3	4	5	6	7	8	9	10	11	12	All patients
*Sex*	M	F	F	M	F	F	F	F	F	M	F	F	
*Age range*	Late adolescence	Adult	Late adolescence	Early adolescence	Middle childhood	Late adolescence	Early childhood (Death)	Early adolescence (Death)	Adult	Late adolescence (Death)	Middle childhood	Adult	
*Molecular diagnosis*	c.1498A > G	c.1486C > T	c.1498A > G	c.1498A > G	c.1498A > G	c.1499 T > C	c.1498A > G	c.1498A > G	c.1498A > G	c.1498A > G	c.1498A > G	c.1499T > C	
*New case*	−	+	+	+	−	−	+	−	+	−	+	−	6/12
*Growth*													
IUGR	+	+ (− 2SD)	+ (− 2SD)	+ (− 2SD)	+ (− 2SD)	+	+ (− 3SD)	+ (− 3SD)	+	+	+ (− 3SD)	+ (− 4SD)	12/12 (100%)
Growth retardation	+ (− 2SD)	+ (− 3,5SD)	+ (− 3,5SD)	+ (− 4SD)	+ (− 3SD)	+ (− 2SD)	+ (− 4SD)	+ (− 4SD)	+	+ (− 2SD)	+ (− 3SD)	+ (− 4SD)	12/12 (100%)
Overweight	−	+ (MC)	+ (EA)	−	−	+	−	−	+	−	−	−	4/12 (33%)
Pseudo-muscular build	+	−	+ (MC)	+	+ (EC)	+	−	+ (MC)	−	+	+ (MC)	+ (MC)	9/12 (75%)
*Facial features*													
Maxillar hypoplasia	−	+ (MC)	+ (MC)	+ (EC)	+ (EC)	+	−	+	+	+	NA	+ (MC)	9/11 (82%)
Short philtrum	+	−	+ (MC)	−	+	−	+	−	−	+	+	+ (MC)	7/12 (58%)
Prognathism	+	+ (MC)	+ (MC)	+ (EC)	+ (EC)	+	−	+	+	+	+	+ (MC)	11/12 (92%)
Small mouth	+	−	−	−	+	+	+	−	−	+	−	−	5/12 (42%)
Fine superior lip	+	−	−	+ (EC)	+ (EC)	−	+	+	NA	+	NA	−	6/10 (60%)
Velar insufficiency	NA	−	−	−	−	NA	−	−	−	−	−	+ (MC)	1/10 (10%)
Short neck	NA	−	−	−	−	NA	+	+	−	NA	−	+ (MC)	3/9 (33%)
Broad nasal bridge	NA	−	+	+ (EC)	−	NA	+	+	+	NA	+	−	6/9 (67%)
Thickened eyebrows	NA	+ (MC)	−	+ (EC)	+ (EC)	NA	−	−	−	NA	−	−	3/9 (33%)
Narrow palpebral fissure	+	+ (MC)	+	−	+ (EC)	+	+	+	−	+	−	+ (MC)	9/12 (75%)
Small ears	+	+ (MC)	+ (MC)	+ (EC)	−	+	+	+	NA	+	−	−	8/11 (73%)
Low ears	NA	+ (MC)	+ (MC)	+ (EC)	−	NA	−	−	NA	NA	−	+ (MC)	4/8(50%)
*Sensory system*													
Refractive disorders	−	+	+	+	+ (EC)	+	+	+	+	+	−	−	9/12 (75%)
Cataract	−	−	−	+	−	+	−	−	−	+	−	−	3/12 25%)
Strabismus	−	+	−	+	+	−	+	+	NA	−	−	−	5/11 (45%)
Hearing impairment	−	−	−	+ (EC)	+ (EC)	+	−	+ (MC)	−	+	+ (MC)	+ (EC)	7/12 (58%)
*Skeletal features*													
Enlarged vertebral pedicles	−	+	+ (MC)	+	NA	+	−	+	NA	+	−	+	7/10 (70%)
Cone-shaped epiphysis	NA	−	+ (MC)	+	+ (EC)	NA	−	−	NA	NA	−	+	4/8 (50%)
Iliac wing hypoplasia	NA	+	+ (MC)	+	−	NA	−	−	NA	+	−	+	5/9 (56%)
Thickened calvarium	NA	NA	+ (MC)	NA	NA	+	−	+	NA	+	−	+	5/7 (71%)
Platyspondyly	NA	−	+ (MC)	−	NA	NA	−	−	NA	−	−	−	1/8 (13%)
Joints limitations	NA	+ (A)	+ (MC)	+ (EC)	+ (EC)	NA	+ (EC)	+ (MC)	−	NA	+ (MC)	+ (MC)	8/9 (89%)
Broad ribs	NA	+	+ (MC)	−	−	NA	−	+	NA	+	−	−	4/9 (44%)
Short long bone	NA	NA	+ (MC)	−	−	NA	+ (EC)	+	NA	NA	−	NA	3/6 (50%)
Short femoral neck	NA	+	+ (MC)	−	−	NA	−	+	NA	+	−	+	5/9 (56%)
Brachydactyly	+	+ (MC)	+ (MC)	+	+ (EC)	NA	+ (EC)	+	+	+	+ (MC)	+	11/11 (100%)
2–3 toes syndactyly	NA	−	−	−	−	NA	−	+	+	NA	−	−	2/9 (22%)
Clinodactyly	NA	−	+ (MC)	−	−	NA	−	+	NA	NA	+ (MC)	+	4/8 (50%)
Camptodactyly	NA	−	−	+ (EC)	+ (EC)	NA	−	−	NA	NA	−	−	2/8 (25%)
*Cardiovascular system*													
Patent arterial duct	−	NA	+	−	−	NA	−	+	−	NA	−	+	3/9 (33%)
Pericarditis	+	NA	−	+ (EC)	−	NA	−	−	−	NA	−	−	2/9 (22%)
Coarctation of the aorta	−	NA	−	+ (Shone)	−	NA	−	−	−	NA	+ (Shone)	−	2/9 (22%)
Systemic hypertension	−	+	−	−	−	+	−	−	+ (A)	NA	−	−	3/11 (27%)
Septal defect	−	NA	−	−	−	−	+ (VSD)	+ (ASD)	−	+	−	−	3/11 (27%)
Aortic stenosis	−	NA	−	+	−	NA	−	−	−	+	+	−	3/10 (30%)
PH	+	NA	+	+	−	NA	NA	+	−	NA	+	−	5/8 (63%)
Vascular stenosis	−	−	−	−	+	NA	NA	+	+	NA Mesenteric ischemia	NA	NA	3/7 (43%)
*Neuro-developmental*													
Microcephalia	NA	+ (− 2SD)	+ (− 2,5SD)	−	−	−	+ (− 3SD)	−	NA	−	+ (− 2SD)	−	4/10 (40%)
Macrocephalia	NA	−	−	+ (+ 2,5SD)	−	−	−	−	NA	−	−	+ (+ 2,5SD)	2/10 (20%)
Intellectual deficit	+ (severe)	−	+ (middle)	+ (middle)	+ (middle)	+	−	+ (middle)	−	+	+ (middle)	+ (middle)	9/12 (75%)
Neurodevelopmental delay	+	−	+ (T)	+ (T)	+	+	−	+ (EC)	−	+	+ (T)	+	9/12 (75%)
Behaviors disorders	NA	−	−	+ (EC)	+ (EC)	NA	−	+ (MC)	−	NA	−	−	3/9 (33%)
Learning difficulties	+	+ (MC)	+ (EC)	+ (EC)	+ (EC)	NA	−	+ (EC)	−	NA	+ (EC)	+	8/10 (80%)
Autistic-like disorders	NA	−	−	−	+ (EC)	NA	−	−	−	NA	−	−	1/9 (11%)
Pathologic brain MRI	NA	−	−	−	−	−	NA	−	+ moyamoya	NA	NA	−	1/8 (13%)
*Respiratory system*													
Respiratory insufficiency	+ (EC)	−	−	−	−	NA	−	+ (EC)	−	NA	−	−	2/10 (20%)
Laryngeal or tracheal stenosis	−	−	−	−	−	−	−	+ (EC)	+ (A)	−	−	−	2/12 (17%)
Pleurisy	+	−	−	+	−	+	+	+	−	NA	+	NA	6/10 (60%)
Asthma	+	+	−	−	−	NA	−	+	NA	NA	−	−	3/9 (33%)
OSA	NA	NA	NA	+	NA	NA	NA	+	NA	NA	+	+	4
*Digestive system*													
Digestive stenosis	−	−	−	−	−	−	+ Esophageal atresia	+ Duodenal stenosis	−	−	−	−	2/12 (16%)
*Reproductive system*													
Cryptorchidism	NA	−	−	+	−	NA	−	−	−	NA	−	−	1
Precocious puberty	NA	+ (MC)	+ (MC)	−	+ (MC)	+	−	+ (MC)	+	+	−	+ (MC)	8/11 (73%)
*Others*													
Thickened skin	+	−	+ (MC)	+ (EC)	−	+	−	+ (MC)	+	+	−	+	8/12 (67%)
Hypogammaglobulinemia	+	NA	+	NA	NA	NA	NA	+	NA	NA	NA	+	4

*NA* no available data, *SD* standard deviation, *PH* pulmonary arterial hypertension, *IUGR* intra-uterine growth retardation, *MRI* magnetic resonance imaging, *OSA* obstructive sleep apnea, *M* male, *F* female, *y/o* years old, *ASD* atrial septal defect, *VSD* ventricular septal defect

*Age range of onset* (*T*) toddler, (*EC*) early childhood, (*MC*) middle childhood, (*EA*) early adolescence, (*LA*) late adolescence, (*A*) adult

## Discussion

The follow up of MS through age emphasizes diagnostic difficulties in newborn and infancy. Indeed, the most specific facial features (prognathism, maxillar hypoplasia, narrow palpebral clefts) were not identified before 2 y/o mainly because the median age of onset of facial characteristics is 6 y/o. In terms of radiological signs, although distal anomalies such as brachydactyly can be detected early, they are nevertheless not sufficient to guide the diagnosis and axial skeletal features suggestive of MS (enlarged vertebral pedicles, thickened calvarium, cones shape epiphyses) are not identified before 6 years. Pseudo-muscular build and thickened skin are specific signs of MS, but also appear around the age of 6 in our cohort. Thus, we had a median age of suspicion for diagnosis at 12 years and molecular confirmation at 14 years. Our results are in agreement with Garavelli et al., who reported no diagnosis before 2 y/o and only 8 patients diagnosed before 8 y/o in a review of 69 subjects published in the literature. The authors also confirm the absence of main clinical and radiological features described above in the first year of life, and an age of onset of the main specific characteristics around 7 y/o [23].

Intellectual disability was present in most patients with delay in psychomotor and language acquisition starting at the age of 2 y/o. We did not identify on medical reports neurodevelopmental disorders before this age, in particular no hypotonia, nor pediatric feeding difficulties. The severity of the delay is heterogeneous, but moderate in most cases. Our long-term follow-up made possible to report 2 adult patients integrated on a socio-professional level. We reported also behavioral disorders, autistic-like disorders as well as psychiatric manifestations, thus joining the data in the literature [15]. It is important to note the onset of progressive deafness which may begin at 2 years in some patients, suggesting the need for regular hearing monitoring.

We confirm the severity of cardiovascular involvement in MS; the two patients with a Shone’s complex were operated on multiple times with complications such as recurrent pericarditis. Among the three deaths that occurred in our cohort, two were related to a cardiovascular origin (PH crises and mesenteric ischemia). These findings are in line with Lin et al., reporting congenital heart disease, cardiomyopathy, systemic hypertension and pericarditis eventually leading to a fatal outcome [24]. The assessment of vascular system revealed stenosis in large and medium vessel in more than a third of the subjects and we identified in a patient a severe moyamoya syndrome, which has not been yet reported in MS. The patient who died from mesenteric ischemia has already been reported by Michot et al. [4], without any etiological explanation for this death, but we can hypothesize that a mesenteric artery stenosis could precede this fatal issue. This stenosing phenotype aspect of MS is also found in the respiratory (airway stenosis, obstructive syndrome) and digestive systems (esophageal atresia, duodenal stenosis).

From the natural history described in our study, we suggest some keys points for the follow up of MS. In newborn and infancy, diagnosis probability is very low, the clinical signs are not specific. Emphasis must be put on the assessment and management of malformations in preschool children, importance should be given to neurodevelopment and hearing assessment. We need also to pay attention to recurrent infections that may suggest immunodeficiency. Specific morphological features are not obvious and clinical suspicion remains low, but with the development of routine exome sequencing, we will undoubtedly have more patients diagnosed in early childhood in the future. In school age children, dysmorphic facial features appear more recognizable, growth retardation associated to radiological signs make the clinical suspicion easier. Joint limitations and thickened skin are very specific of MS and onset in this period. It is also possible to detect vascular stenosis, but they are mostly non-symptomatic. The adolescence is marked by obstructive respiratory disease which could be complicated with PH and vascular stenosis is also expressed at this period. Specialized monitoring should be rigorous because their complications are potentially fatal. Moreover, cardiovascular risk factors such as systemic arterial hypertension, diabetes and overweight could occur at the same time. On adulthood, patients may encounter primary infertility. However, Meerschaut et al. [17], reported for the first-time pregnancy in MS patient, but only by assisted reproductive treatment, the patient gave birth to two affected children.

The pathophysiology of MS remains unclear to this day. The presence of multiple congenital abnormalities is correlated with the expression pattern of *SMAD4*, which is expressed ubiquitously in embryonic development and in most adult tissues and cells [33]. *SMAD4* mediates TGFβ/BMP which plays an important role in tissue regeneration, cell differentiation, embryonic development and regulation of the immune system [34, 35]. Immune deficiency and damage to the serous membranes (pericarditis and pleurisy) are found recurrently in our cohort and in the literature, which clearly highlights the role of *SMAD4* in the immune system.

Mouse models have been developed and homozygous activation of *SMAD4* leads to the death of embryo: moreover mutated embryos are smaller in size than wild embryos. Targeted inactivation of *SMAD4* in mouse chondrocytes results in dwarfism with histological abnormalities in growth plate associated with sensorineural hearing loss due to size and histological abnormalities of the cochlea [36]. This confirms the role of *SMAD4* in the development of the inner ear and partly explains the development of deafness in some patients with MS. The targeted inactivation of *SMAD4* in mouse osteoblasts has been shown to be involved in postnatal bone homeostasis as well. This function could explain the bone abnormalities and facial features (especially prognathism and maxillar hypoplasia) observed in patients with Myhre syndrome [37].

*SMAD4* is also known to be a tumor suppressor gene. Indeed, mutations leading to loss of function of *SMAD4* have been reported in juvenile polyposis syndrome, characterized by the presence of polyps in the digestive tract in children and an increased rate of colorectal cancer [38, 39]. No tumor was observed in our cohorts but Lin et al. [21], reported in a series of 61 patients 6 patients with neoplasia and warned cancer susceptibility in MS.

In our cohort, we noticed that mutations affecting Ile500 lead to severe clinical spectrum including moyamoya syndrome (n = 1), PH (n = 5), Shone’s complex (n = 2), pericarditis (n = 2), vascular (n = 2), digestive (n = 2) and respiratory tract (n = 2) stenosing. Moreover, the three patients with fatal issue had Ile500Val mutations. By contrast, the patient with mutation affecting Arg486 did not have any life threatening complication. Similarly, three patients with Arg486 reported by Caputo et al. [3] and Michot et al. [4] did not have stenosing diseases or congenital heart malformation. Despite a limited number of patients, we hypothesized a possible correlation between genotype and phenotype.

To date, biomarkers assessing the progression of the disease and targeted therapeutics are under investigation. Piccolo et al. [40], showed that losartan normalizes the transcript levels of metalloproteinase-related inhibitors and corrects deposition abnormalities in the extracellular matrix of fibroblasts from patients with MS. Recently, Cappuccio et al., lead a pilot clinical trial with losartan on four MS patients and identified clinical endpoints on skin thickness, joints range of motion and speckle-tracking echocardiogram of left ventricular function to evaluate losartan efficacy. Authors suggested that losartan might provide benefit to patient with improvements in skin thickness, joint range of motion and to a lesser extent of myocardial strain in MS [41]. The natural history of MS provided by our study highlighted the occurrence of life-threatening complications such as vascular stenosis or PH. These endpoints should be therefore also considered to establish the efficacy of novel therapies.

Our study is a retrospective study and has therefore several limitations related to the design of the study. First, there is a follow-up bias based on the availability of data. There is also a measurement bias because our study is based on a review of medical records which are filled in heterogeneously depending on the physician. It is possible that a clinical sign is present before notification in the medical reports and the lack may also be due to an oversight or ignorance of the practitioner during the clinical examination. The severity of MS is quite variable among the patient. Our study being carried out in a single center in a pediatric university hospital specializing in genetic diseases selection bias is also possible considering that the most severe patients are concentrated in a tertiary hospital.

## Conclusion

The study of natural history of a rare disease is confronted with 2 issues, a limited number of cases and a heterogeneity of the clinical presentation. An exhaustive description of the entire clinical spectrum leads to a long and complex data collection. By chaining information from medical report, medical imaging and a secondary using of electronic health record, we assess natural history of MS and describe an evolving disease with multisystemic disorders from newborn to adulthood, requiring multidisciplinary monitoring. We highlighted the occurrence of potentially fatal complications such as vascular stenosis or PH, underlining the severity of the disease and the need to implement recommendations for diagnostic management and therapeutic. To validate these results, a prospective study of the natural history is necessary.

## Data Availability

The datasets generated and analyzed during the current study are not publicly available due to data privacy reasons but are available from the corresponding author upon reasonable request.

## Abbreviations

MS: Myhre syndrome
y/o: Years old
NEM: Necker-Enfants Malades University Hospital
RC RSD: Reference center for rare skeletal dysplasia
HPO: Human Phenotype thesaurus
DrWH: Dr Warehouse
IUGR: Intra uterine growth retardation
SD: Standard deviation
PH: Pulmonary arterial hypertension
MRI: Magnetic resonance imaging
TGFβ: Transforming growth factor beta
BMP: Bone morphogenetic protein
